# Toll-Like Receptor Signaling and Its Role in Cell-Mediated Immunity

**DOI:** 10.3389/fimmu.2022.812774

**Published:** 2022-03-03

**Authors:** Tianhao Duan, Yang Du, Changsheng Xing, Helen Y. Wang, Rong-Fu Wang

**Affiliations:** ^1^ Department of Medicine, Keck School of Medicine, University of Southern California, Los Angeles, CA, United States; ^2^ Department of Pediatrics, Children’s Hospital Los Angeles, Keck School of Medicine, University of Southern California, Los Angeles, CA, United States; ^3^ Norris Comprehensive Cancer Center, Keck School of Medicine, University of Southern California, Los Angeles, CA, United States

**Keywords:** toll-like receptors, cell-mediated immunity, T cells, signaling pathway, infectious diseases, autoimmune diseases, cancer

## Abstract

Innate immunity is the first defense system against invading pathogens. Toll-like receptors (TLRs) are well-defined pattern recognition receptors responsible for pathogen recognition and induction of innate immune responses. Since their discovery, TLRs have revolutionized the field of immunology by filling the gap between the initial recognition of pathogens by innate immune cells and the activation of the adaptive immune response. TLRs critically link innate immunity to adaptive immunity by regulating the activation of antigen-presenting cells and key cytokines. Furthermore, recent studies also have shown that TLR signaling can directly regulate the T cell activation, growth, differentiation, development, and function under diverse physiological conditions. This review provides an overview of TLR signaling pathways and their regulators and discusses how TLR signaling, directly and indirectly, regulates cell-mediated immunity. In addition, we also discuss how TLR signaling is critically important in the host’s defense against infectious diseases, autoimmune diseases, and cancer.

## Introduction

The innate immune system is the first line of defense against infectious pathogens and cancer by sensing and responding to the structure-conserved molecules of the pathogens (pathogen-associated molecular patterns, or PAMPs) as well as the endogenous ligands released from damaged cells (damage-associated molecular patterns, or DAMPs). The pattern recognition receptors (PRRs) are a key element of the immune system, including Toll-like receptors (TLRs), RIG-I-like receptors, Nod-like receptors (NLRs), AIM2-like receptors, C-type lectin receptors, and intracellular DNA and RNA sensors (1–3). Upon the recognition of their specific ligands from the invasive pathogens or damaged cells, PRRs initiate a variety of downstream signaling cascades, including nuclear factor kappa B (NF-κB), type I interferon (IFN) and inflammasome signaling pathways, leading to the production of corresponding proinflammatory or antiviral cytokines and chemokines (2, 4). The activation of TLR signaling is also crucial to the induction of antigen-specific adaptive immune responses by promoting the maturation of dendritic cells (DCs) and activating the adaptive immune cells for the clearance of invading pathogens (4–6).

TLRs belong to the family of Type I integral membrane glycoproteins characterized by the extracellular domains containing variable numbers of leucine-rich-repeat (LRR) motifs and a cytoplasmic Toll/interleukin 1 (IL-1) receptor (TIR) homology domain (7). Toll was identified initially as a gene controlling dorsoventral axis formation of the *Drosophila* embryo in the 1980s (8), and its crucial anti-fungal function in *Drosophila* was demonstrated in 1996 (9). The mammalian homolog of the Toll receptor (now termed TLR4) was first discovered in 1997 to play a critical role the in innate immunity by inducing the expression of inflammatory responses-related genes (10). These findings revolutionized our understanding of the immune system and triggered an explosion of research in PRRs. To date, 10 TLRs have been identified in humans (TLR1–TLR10) and 12 in mice (TLR1–TLR9 and TLR11–TLR13). TLR1–TLR10 are conserved between mice and humans, although mouse TLR10 is not functional, while TLR11–TLR13 are expressed only in mice but not in humans. These receptors are localized on the cell surface (TLR1, TLR2, TLR4, and TLR5) or in intracellular compartments, such as the endoplasmic reticulum, endosome, lysosome, or endolysosome (TLR3, TLR7, TLR8, and TLR9) (6).

Cell surface TLRs mainly recognize membrane components of the microorganisms such as lipids, lipoproteins, and proteins (2). For example, TLR4 recognizes lipopolysaccharide (LPS). TLR2 forms a heterodimer with either TLR1 or TLR6 and recognizes different PAMPs of pathogens (including lipoproteins, peptidoglycans, lipoteichoic acids, zymosan, mannan, and glycosylphosphatidylinositol-anchored mucin-like glycoproteins from Trypanosoma cruzi trypomastigotes) (11). TLR5 recognizes the flagellin of bacteria (2). Human TLR10 can homodimerize or heterodimerize with TLR1, TLR2, and TLR6 (12), and sense HIV proteins (13). Intracellular TLRs mainly recognize nucleic acids derived from pathogens or self-nucleic acids in a disease condition. TLR3 recognizes double-stranded viral RNA and self RNAs derived from damaged cells; TLR7, TLR8, and TLR13 recognize fragments of single-stranded RNA with distinct sequence preferences, and TLR7 is predominantly expressed in plasmacytoid dendritic cells (pDCs). In addition, TLR9 recognizes single-stranded DNA containing unmethylated cytidine-phosphateguanosine (CpG) motifs from bacteria or viruses (6, 14). TLR10 was recently identified to sense HIV-1 gp41 protein but its biological functions in humans haven't been fully elucidated (12, 13).

Each TLR contains a similar cytoplasmic portion known as the TIR domain, which is highly similar to that of the IL-1 receptor family. The extracellular portion of TLRs is the ectodomain, with LRRs displayed as a horseshoe-like structure. The characteristic feature of these LRRs is the consensus sequence motif—L(X_2_)LXL(X_2_)NXL(X_2_)L(X_7_)L(X_2_)—in which X can be any amino acid (15). The ectodomain of TLRs forms a homo- or hetero-dimer along with a co-receptor or accessory molecule to interact with their respective PAMPs or DAMPs (16). TLRs are expressed on all the innate immune cells and a large majority of non-hematopoietic cells, such as macrophages, neutrophils, DCs, natural killer cells, mast cells, basophils, eosinophil, and epithelial cells (4, 17). Importantly, TLRs can also be detected on adaptive immune cells, including T and B cells (18, 19).

Adaptive immunity consists of humoral immunity and cell-mediated immunity, which are mainly mediated by B lymphocytes and T lymphocytes, respectively. Cell-mediated immunity (also called cellular immunity) is responsible for generating a cluster of differentiation 8 (CD8)^+^ cytotoxic T-lymphocytes (CTLs) and an antigen-specific cluster of differentiation 4 (CD4)^+^ T helper (Th) cells, which help B cells produce antibodies. CTLs recognize and produce molecules that directly kill infected host cells. In contrast, Th cells release various cytokines that influence the function of other cells involved in both adaptive and innate immune responses (20). To induce efficient activation and clonal expansion of antigen-specific T cells, antigen presentation and co-stimulatory signaling are essential, which must be simultaneously provided by the antigen-presenting cells (APCs) to T cells. Importantly, the production of cytokines, expression of costimulatory molecules, and antigen-presenting activity in APCs are induced or enhanced by microbe-derived adjuvants, which are recognized by TLRs expressed on APCs and boost the APC signaling to promote activation of immune responses in T cells (21). Therefore, TLRs play a critical role in linking the innate immunity and cell-mediated immunity. This review article mainly summarizes the recent progress on TLR signaling pathways and their crucial role in cell-mediated immunity.

## TLR Signaling Pathways

Innate immunity was formerly thought to be a nonspecific immune response. However, the discovery of TLRs led to the realization of the considerable specificity of innate immunity and its capability to discriminate between self and nonself (22–24). Cell surface TLRs (TLR1, TLR2, TLR4, TLR5, TLR6, and TLR11) mainly recognize microbial membrane components to induce an inflammatory response (11). By contrast, intracellular TLRs (TLR3, TLR7, TLR8, and TLR9) mainly recognize microbial nucleic acids derived from bacteria or viruses and induce Type I IFN responses and inflammatory responses. However, the misrecognition of self-nucleic acids may cause autoimmune diseases (25).

Upon binding by specific ligands, ligand-mediated dimerization of TLR ectodomains results in the coordinate dimerization of the cytosolic TIR domains of each TLR (26). Dimerized receptor TIR domains are detected by two receptor-proximal membrane adaptor proteins: the TIR domain-containing adapter protein (TIRAP; also known as MAL) (27, 28) and the TIRAP-inducing IFN-β (TRIF)-related adaptor molecule (TRAM) (29, 30). These peripheral membrane proteins survey the inner leaflets of the plasma and endosomal membranes through the actions of an N-terminal phosphoinositide binding domain of TIRAP or a bipartite localization domain of TRAM consisting of an N-terminal myristoylation motif and a phosphoinositide-binding motif (31–33).

**Figure 1:**
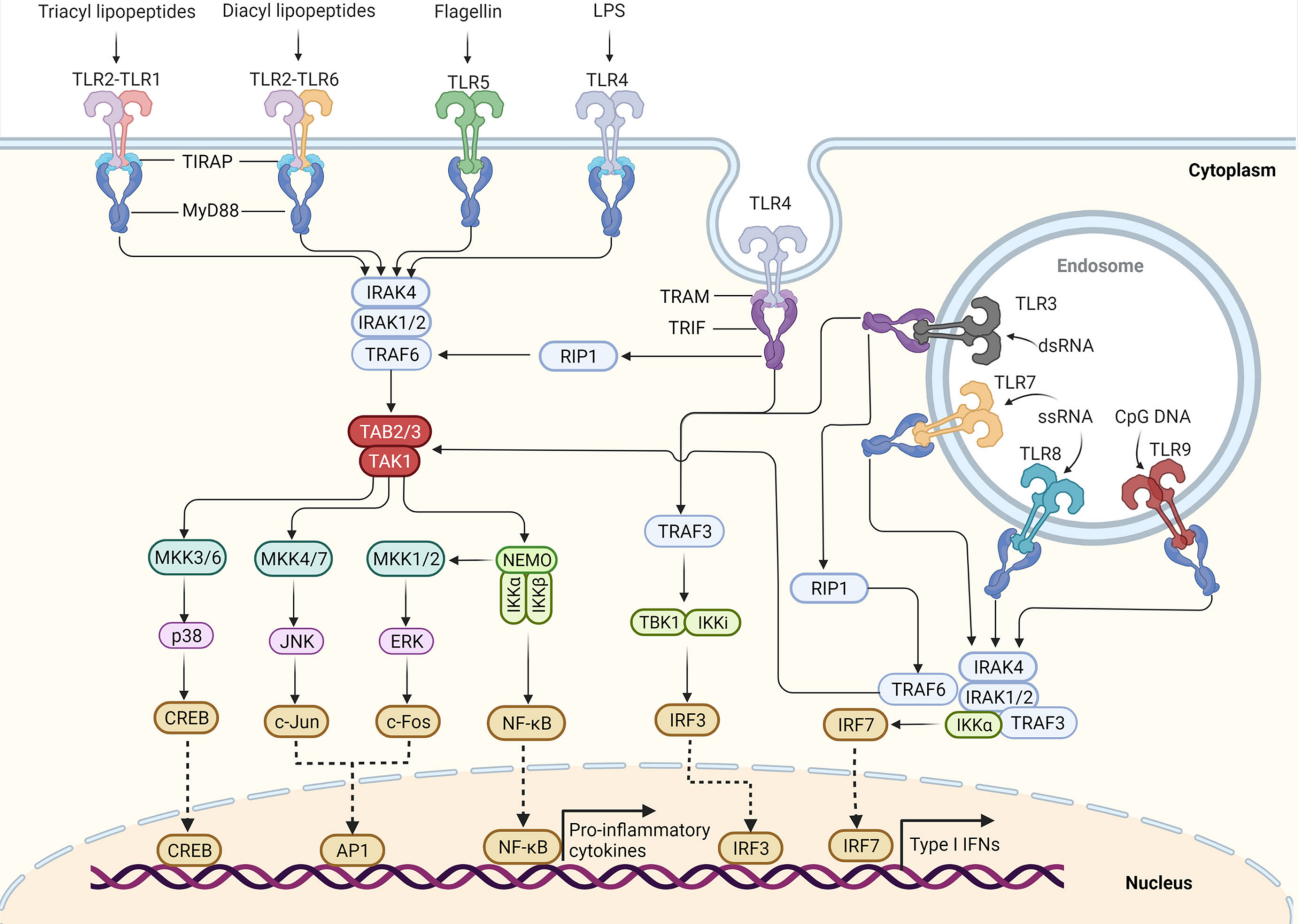
TLR signaling pathway in innate immune cells. TLR5, TLR4, and the heterodimers of TLR2–TLR1 or TLR2–TLR6 prefer to recognize the membrane components of pathogens at the cell surface, whereas TLR3, TLR7–TLR8, and TLR9 localize to the endosomes, where they recognize the nucleic acids from both the host and foreign microorganisms. TLR4 localizes at the plasma membrane, but it is endocytosed into endosomes upon activation. Upon binding to their respective ligands, TLR signaling is initiated by dimerization of receptors, leading to the engagement of TIR domains of TLRs with TIRAP and MyD88 (or directly interact with MyD88) or with TRAM and TRIF (or directly interact with TRIF). The TLR4 signaling switches from MyD88 to TRIF once TLR4 moves to the endosomes. Engagement of MyD88 recruits the downstream signaling molecules to form Myddosome, which is based on MyD88 and contains IRAK4 and IRAK1/2. IRAK1 further activates the E3 ubiquitin ligase-TRAF6 to synthesize the K63-linked polyubiquitin chains, leading to the recruitment and activation of the TAK1 complex. The activated TAK1 further phosphorylates and activates the canonical IKK complex, ultimately leading to the activation factor NF-κB. The activation of TAK1 also leads to the activation of MAPKs, including MKK4/7 and MKK3/6, which further activate JNK and p38, respectively. The activation of IKKβ also leads to the activation of MKK1 and MKK2, which further activate ERK1/2. The activation of these MAPKs leads to some important transcription factor activations, such as CREB, AP1. These transcription factors cooperate with NF-κB to promote the induction of pro-inflammatory cytokines. Engagement of TRIF recruits the TRAF6 and TRAF3. Activated TRAF6 can recruit the kinase RIP1 and activate the TAK1 complex and IKK complex, leading to the activation of NF-κB and MAPKs. TRIF also promotes the TRAF3-dependent activation of the TBK1 and IKKϵ (originally IKKi), which further phosphorylates and activates IRF3. Among TLR7, TLR8, and TLR9 signaling in pDCs, IRF7 can bind to the Myddosome and is directly activated by IRAK1 and IKKϵ. Activation of IRF3 and IRF7 leads to the induction of Type I IFN.

TIRAP and TRAM can further recruit myeloid differentiation primary response protein 88 (MyD88) and TRIF, respectively (34), and stimulate the assembly of a large oligomeric scaffold called Myddosome or Triffosome (35). These supramolecular complexes consist of downstream signaling components and kinase enzymes. Increased local concentrations of signaling molecules promote the intrinsically weak allosteric interactions and initiate cytosolic signaling transduction (36). Depending on the distinct supramolecular complexes formed, TLR signaling pathways can be mainly classified as either MyD88-dependent pathways, which drive the induction of inflammatory cytokines, or TRIF-dependent pathways, which are responsible for the induction of Type I IFN as well as inflammatory cytokines (2) **(** **Figure 1** **)**.

## MyD88-Dependent Pathway

MyD88 is the first identified member of the TIR family; it is commonly used by all the TLRs except TLR3, and it activates the NF-κB signaling pathway (11). Upon activation by specific ligands, MyD88 recruits IL-1 receptor-associated kinases (IRAK)—IRAK4, IRAK1, IRAK2, and IRAK-M—which form a complex with IRAK kinase family members, referred to as the Myddosome (37–39). During Myddosome formation, IRAK4 is activated initially by MyD88 through its N-terminal death domain, which is also contained in IRAK4. Similar to MyD88, IRAK4 is also essential for the activation of NF-κB and mitogen-activated protein kinases (MAPKs) in the MyD88-dependent pathway (40, 41). The activated IRAK4 can sequentially activate IRAK1 and IRAK2, which are then autophosphorylated at several sites (42). Although activation of both kinases is required for robust activation of TLR-induced NF-κB and MAPK signaling, the relative importance of IRAK1 and IRAK2 may differ in humans and mice (43).

Activated IRAK1 can interact with tumor necrosis factor (TNF) receptor-associated factors 6 (TRAF6), an E3 ligase that catalyzes the synthesis of Lys63 (K63)-linked polyubiquitin, resulting in activation of TRAF6. TRAF6, along with E2 ubiquitin-conjugating enzymes Ubc13 and Uev1A, generates the K63-linked polyubiquitin chains and promotes K63-linked polyubiquitination of both TRAF6 itself and IRAK1. Early studies suggested that K63-linked polyubiquitination of TRAF6 and IRAK1 might serve as a platform for activation of downstream TGFβ-activated protein kinase 1 (TAK1) or IκB kinase (IKK) (44–48). However, the direct biochemical evidence is missing and the conflicting results have been reported that ubiquitination of TRAF6 may be dispensable for the downstream protein kinase activation (49). Therefore, whether the K63-linked polyubiquitination of TRAF6 and IRAK1 can directly activate downstream protein kinases or it just serves as a marker of signaling pathway activation still requires further investigations. Recent biochemical studies revealed that the free K63 polyubiquitin chains synthesized by TRAF6 and Ubc13/Uev1A, which are not conjugated to any cellular protein, could directly activate TAK1 *in vitro* by binding to the novel zinc finger-type ubiquitin-binding domain of TAB2 and TAB3 (50), leading to close proximity-dependent transphosphorylation of TAK1 at Thr-187 (50, 51). However, whether and how these free polyubiquitin chains activate downstream protein kinases *in vivo* remains to be determined. Phosphorylated TAK1 then activates the IKK complex-NF-κB pathway and -MAPK pathway (6).

The IKK complex is comprised of the catalytic subunits IKKα and IKKβ and the regulatory subunit nuclear factor-κB essential modulator (NEMO) (also called IKKγ) (44). K63 polyubiquitin chains might bridge TAK1 to form a complex with IKK, thus allowing TAK1 to phosphorylate IKKβ through its close proximity to the IKK complex, which leads to activation of the IKK complex (52–54). Recently, Met1-linked ubiquitin dimers (also known as linear ubiquitin dimers) were shown to bind with 100-fold higher affinity to NEMO compared with K63-linked ubiquitin (55, 56), indicating that linear ubiquitination, catalyzed by the linear ubiquitin chain assembly complex (LUBAC), also contributes to the activation of IKK (57–63). The activated IKK complex can further phosphorylate the NF-κB inhibitory protein IκBα, which undergoes proteasome degradation, allowing NF-κB to translocate into the nucleus to induce proinflammatory gene expression (6).

In the MAPK pathway, the activated TAK1 simultaneously activates the MAPK family members Jun N-terminal kinases (JNKs) and p38 by inducing the phosphorylation of MAPK kinases 4/7 (MKK4/7) and MKK3/6. The IKKβ also catalyzes the phosphorylation of p105 to cause its degradation by the Skp1-Cul1-F-box ubiquitin ligase (SCF^βTrCP^) complex, producing p50 and releasing tumor progression locus 2 (TPL2) to activate MKK1/2, which further phosphorylates and activates extracellular signal−regulated protein kinase 1 (ERK1) and ERK2. These MAPKs then phosphorylate cyclic AMP-responsive element-binding protein (CREB) and activator protein 1 (AP-1) transcription factors consisted of a heterodimer of c-Fos and c-Jun subunits to regulate inflammatory responses (44). TAK1 is a central component of MyD88-dependent NF-κB and MAPK signaling pathways. An earlier study suggested that TAK1 is required for the activation of the NF-κB and MAPK signaling pathway in both mouse embryonic fibroblast cells, B cells and T cells (64–68). However, we found that TAK1 serves as a negative regulator in mouse neutrophils (69, 70). By contrast, TAK1 might serve as a positive regulator in human neutrophils (71), suggesting a cell type-specific role for TAK1 in TLR-induced signaling (72). Interestingly, we recently found that *Tak1* deficiency in mice alters the intestinal microbiome, which can drive protective immunity against colitis and colorectal cancer (73).

Among TLR7, TLR8, and TLR9 signaling in pDCs, MyD88 also activates NF-κB signaling and interacts with interferon regulatory factor (IRF)-5 and IRF-7 for the induction of proinflammatory cytokines or Type I IFN (IFN-α and IFN-β) responses (74–76). IRF7 is highly expressed by pDCs, which can bind to the Myddosome containing IRAK4, TRAF6, TRAF3, IRAK1, and IKKα (77). IRAK1 and IKKα further phosphorylate the IRF7 protein, leading to its dissociation from the Myddosome and dimerization. The IRF7 homodimer translocates into the nucleus and drives IFNα expression (11). By contract, IRF5 is phosphorylated by IKKβ on Ser462 and contributes to proinflammatory cytokine transcription but not IFNα production (76, 78–80).

## TRIF-Dependent Pathway

In macrophages and conventional DCs (cDCs), TLR3- or TLR4-induced IFN expression is not dependent on MyD88 but instead is driven by TRIF as well as the proteins TRAM and TRAF3 (29, 30, 81, 82). Upon detection of dimerized TLR4 in endosomes, TRAM is thought to interact with TRIF to induce the formation of the putative Triffosome (35), in which TRIF interacts with TRAF6 and TRAF3. Activated TRAF6 can recruit the kinase receptor-interacting protein 1 (RIP1), which in turn recruits and activates the TAK1 complex and IKK complex, leading to activation of NF-κB and MAPKs and the induction of inflammatory cytokines (6). An earlier study suggested that TRAF6 might mediate RIP1 ubiquitination (83). However, TRAF6 has also been reported to be dispensable for TRIF-dependent TLR signaling (84), suggesting that additional E3(s) might be responsible for RIP1 ubiquitination. Recently, an E3 ubiquitin ligase Peli1 was found to facilitate TRIF-dependent TLR signaling and proinflammatory cytokine production by inducing the ubiquitination of RIP1 (85), indicating that Peli1 might share a redundant role with TRAF6.

TRIF also promotes the TRAF3-dependent activation of the IKK-related kinase TANK-binding kinase 1 (TBK1). TRAF3 activates the TBK1 and inhibitor of NF-κB kinase (IKKi) along with NEMO for phosphorylation and dimerization of the IFN-inducing transcription factor IFN regulatory factor 3 (IRF3). Subsequently, the IRF3 homodimer translocates into the nucleus from the cytoplasm, where it drives the expression of Type I IFN genes and IFN-stimulated genes (ISGs) (86–88). Recently, a 39-amino-acid pLxIS motif was identified within TRIF (but not MyD88), which can be phosphorylated by TBK1. The phosphorylated motif can recruit IRF3, leading to the phosphorylation and activation of IRF3 by TBK1 (89, 90). Therefore, the TLR4 uses TRIF but not MyD88 to promote IRF3-induced IFN expression in the endosome. Unlike TLR4, TRAM cannot interact with TLR3 or regulate TLR3 signaling (29), indicating that TLR3 might directly interact with TRIF or use another sorting adaptor to link TRIF to TLR3.

## TLR-Mediated Regulation of APCs

**Figure 2:**
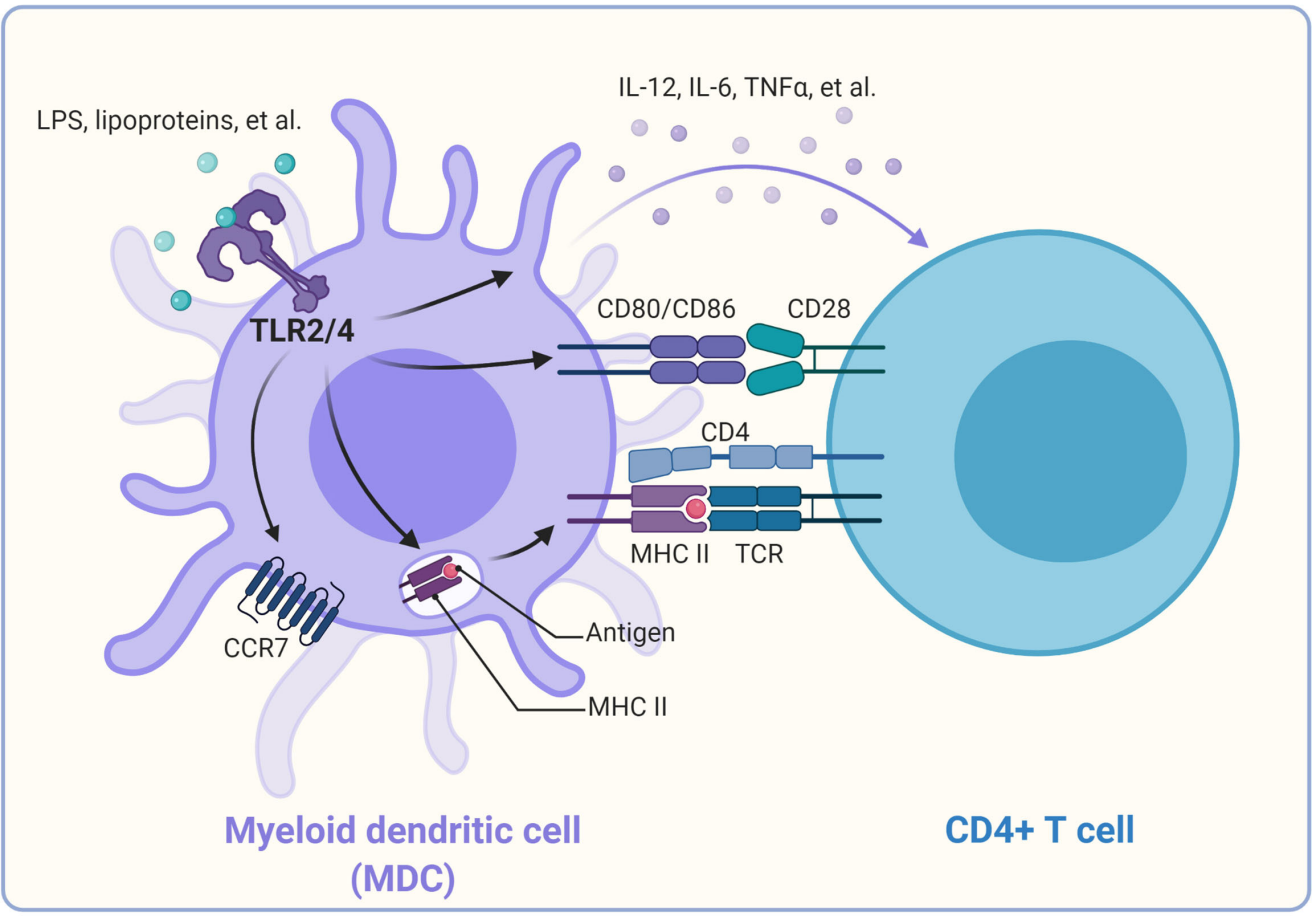
Promotion of CD4^+^ T cell activation by TLRs on dendritic cells. Once TLR2/4 recognize their individual ligands, they can alter the expression of chemokine receptors (CCR2, CCR5, and CCR7), leading to DC migration from the infected tissue to the draining lymph node, where naïve T cells are stimulated. TLR2/4 signaling can promote the antigen process and bind to the major histocompatibility complex II and be presented to the CD4^+^ T cells, thus providing the first signal for activation of the CD4^+^ T cells. In addition, TLR signaling triggers the up-regulation of costimulatory molecules on the cell surface of DCs, which provide the second signal to activate the antigen-specific CD4^+^ T cells. TLR signaling can also induce the production of cytokines such as IL-12, TNF-α in DCs. These cytokines function as “instructive” cytokines and drive the activation and differentiation of CD4^+^ T cells.

TLR-mediated activation and maturation of DCs and macrophages are critical links between innate and adaptive immunity (21). DCs are professional APCs and play a central role in inducing the activation and differentiation of naïve T cells into Th type 1 (Th1) cells, Th2 cells, and CTL effectors (91). Once DCs take up the antigen, the activated DCs can migrate to local lymphoid tissues to present the antigenic peptides on the relevant major histocompatibility complex (MHC) molecules (4). This process is regulated by recognizing pathogens *via* the variety of PRRs expressed by DCs. Among these PRRs, TLR family members play a critical role in generating effector T cell responses (4, 92, 93). The production of “innate” cytokines (type I IFN, IL-1, IL-6, IL-12, TNF-α), up-regulation of costimulatory molecules (CD40, CD80, and CD86), and altered expression of chemokine receptors (CCR2, CCR5, and CCR7) are the characteristics of DC maturation (21), which can be induced by ligands of TLRs, including LPS, lipoproteins, and CpG DNA (4, 75, 81, 94–96) (**Figure 2** **)**.

Moreover, TLR signals can also facilitate peptide loading onto MHC molecules or the cross-presentation of exogenous antigens for the stimulation of CD8^+^ T cell responses by promoting the acidification of endosomes or the fusion of MHC Class I-containing endosomes with phagosomes in DCs (97–99). In addition, LPS-induced TLR signaling can promote the redistribution of MHC Class I and II molecules to the surface of DCs (100). TLR4 activation on DCs promotes cytosolic routing of dendritic cell-specific intercellular adhesion molecule-3-grabbing non-integrin (DC-SIGN)-targeted antigens for presentation on MHC Class I and increased CD8^+^ T cell activation (101). Recently, TLR3, TLR4, and TLR9 ligands were reported to induce autocrine C3a receptor and C5a receptor (C3ar1/C5ar1) signaling in DCs, which causes the expansion of effector T cells and instability of regulatory T cells and contributes to T cell-dependent transplant rejection (102). IFN-γ combined with TLR ligation TLR2, TLR4, or TLR9 agonists can enhance DC activation and function to increase antigen-specific T cell responses (103).

Interestingly, TLR2 seems more critical than TLR4 in mouse DC-derived IL-10 responses to schistosome antigens (104). TLR2 signaling activation on DCs can promote higher frequency effector and memory CD4^+^ T cell responses than TLR4 signaling activation. The novel TLR2 agonist SUP3 also showed a heightened ability to enhance DC-mediated antigen presentation and T cell activation (105). By contrast, the TLR5 ligand flagellin was most effective at activating neonatal lung APCs by inducing significantly higher expression of maturation markers on CD103^+^ (cDC1) and CD11b^+^ (cDC2) subsets (106). Monocyte-derived DCs stimulated with TLR4 and TLR7/8 ligands induce naive allogeneic CD4^+^ T cells to secrete IL-10 and IFN-γ sequentially and eventually IL-17A (107). The activation of TLR9 and IL-12 pathways in CD8α^+^ DCs can drive CD4^+^ T cells to act as Th cells or induce rapid polyclonal conversion to immunosuppressive Treg during *Listeria* infection (108). Interestingly, although all TLRs on DCs are able to induce CD8^+^ T cell activation *in vitro*, the abilities of surface and endosomal TLRs to activate CD8^+^ T cells might be different *in vivo*. The nucleic acid recognizing endosomal TLRs potently induce CD8^+^ T cell activation, whereas the bacterial ligands recognizing surface TLRs were incapable of inducing CD8^+^ T cell priming. Moreover, surface TLRs might have a dominant effect of inhibiting CD8^+^ T cell expansion induced by activation of endosomal TLRs (109).

Based on the particular cell surface markers, DCs can be divided into different subsets, including myeloid DCs, pDCs, CD8α^+^ DCs, and CD11b^+^ DCs (4). Human pDCs express TLR7 and TLR9, whereas CD11c^+^ human myeloid DCs express TLR1, TLR2, TLR3, TLR5, TLR6, and TLR8 (110–112). Human blood monocytes express TLR1, TLR2, TLR4, and TLR5, but progressively lose these receptors and acquire the expression of TLR3 as they differentiate into mature DCs in the presence of granulocyte-macrophage colony-stimulating factor and IL-4 (113). Mice splenic DC subsets express TLR1, TLR2, TLR4, TLR6, TLR8, and TLR9, but not TLR3 (114). Interestingly, freshly isolated mouse splenic DC subsets or macrophages only express low amounts of TLR4 and do not respond to LPS stimulation. By contrast, bone marrow-derived DCs or macrophages have high expression of TLR4 and respond robustly to LPS (115).

Since different DC subsets express subset-specific PRRs, DCs are functionally heterogeneous (110, 114, 116). Different DC subsets respond to different stimuli and activate distinct signaling pathways, leading to the release of specific cytokines, which in turn determine the specific Th cell subsets that are generated and activated (117). pDCs express TLR7 and TLR9, which recognize ssRNA and CpG DNA, respectively, but pDCs do not express other TLRs that detect bacterial cell wall components. Therefore, pDCs are thought to specifically detect viral infections to induce Type I IFNs and control antiviral immunity (35, 116).

## TLR-Mediated Regulation of T Cells

TLR signaling in innate immune cells indirectly regulates T cell differentiation and proliferation by promoting DC maturation and regulatory cytokine production (118). Since T cells also express different TLRs, recent studies have revealed that TLR-mediated signaling can directly regulate effector T cells and Treg cells (119).

CD4^+^ Th cells play a critical role in initiating and maintaining adaptive immune responses against cancer (120). CD4^+^ Th cells are required for the expansion and maintenance of memory CD8^+^ T cells (121). Naïve murine or human CD4^+^ T cells can express TLR2 after their stimulation (122, 123). TLR2 signaling can promote the proliferation and production of IFNγ in Th1 cells (124). Costimulation of TCR and TLR2 in naïve murine CD4^+^ T cells increases their differentiation to proinflammatory Th1 cells and secretion of cytokines and chemokines (122, 125). Costimulation of neonatal CD4^+^ T cells with TLR2 ligand and anti-CD3 also show an increased proinflammatory Th1 immune response (IFN-γ and TNF-α production) and IL-2 production (126). Moreover, TLR2 signaling on CD4^+^ T cells exerts a protective action by increasing the population of *Mycobacterium tuberculosis* Ag-specific T cells during mycobacterial tuberculosis (127).

Similarly, TLR2 signaling can also regulate the immune response of the CD8^+^ T cell. TLR2 agonists can enhance cell survival, proliferation, IFN-γ production, and memory cell formation of CD8^+^ T cells in response to a suboptimal TCR signal by reducing the threshold for costimulatory signals from APCs (128–130). The TLR2/MyD88-dependent signaling pathway in CD8^+^ T cells also can increase their survival, clonal expansion, and differentiation into long-lived memory T cells by activating the phosphatidylinositol 3-kinase (PI3K)-Akt pathway during vaccinia virus infection (130). Interestingly, MyD88-dependent signaling is also essential for CD4^+^ T cell-promoted IFN-γ production and hematopoietic progenitor cell expansion during intracellular bacterial infection (131). In addition, MyD88-dependent signaling in the host can protect against acute allogenic graft versus host disease after bone marrow transplantation (132). However, activating the MyD88 signaling pathway in donor CD4^+^ T cells promotes the survival and differentiation of T cells toward Th1, Tc1, and Th17. It increased the severity of graft versus host disease in a mouse model of allogeneic hematopoietic stem cell transplantation (133). MyD88-dependent signaling is also reported to promote differentiation and proliferation of CD4^+^ T cells toward Th17 cells by linking IL-1 and IL-23 signaling and sustaining mTOR signaling (134).

Besides TLR2, TLR4 is also expressed on CD4^+^ T cells, and the TLR4 ligation could enhance both the *in vitro* cell proliferation and survival of CD4^+^ T cells (135). However, the activation of TLR4 signaling could affect the phenotype and ability of CD4^+^ T cells to provoke the intestinal inflammation, through the induction of MAPK phosphatase 3 (MKP-3) to inhibit TCR stimulation-induced activation of ERK1/2 (136). Moreover, LPS can induce the adhesion of human T cells to fibronectin and the up-regulated expression of suppressor of cytokine signaling 3 (SOCS3), which further led to the inhibition of T cell chemotaxis toward the chemokine stromal cell-derived factor 1α (CXCL12) (137, 138). By contrast, CD4^+^ T cells are pathologic and contribute to an exaggerated immune activation in the mice that is absence of functional Tregs, resulting in the mortality to a nonlethal dose of LPS or *Escherichia coli* challenge (139). Recently, it was reported that the TLR4 expression on T cells goes down during TCR and mitogenic activation (140). However, the VIPER peptide (VP), an established inhibitor of TLR4 signaling, restores TLR4 expression and regulates the activation of naive T cell, indicating that TLR4 responses might be associated with the acute-stage T cell responses (140).

The agonist of TLR9 (CpG-ODNs) was found to promote the release of IL-8 in purified CD8^+^ T cells (110). Additionally, the expression of TLR7 is increased in the mesenteric lymph node CD4^+^ and CD8^+^ T cells after *Schistosoma japonicum* infection. The TLR7 agonist can enhance the production of IFN-γ in CD8^+^ T cells from mesenteric lymph node T cells in infected mice (141). Moreover, TLR7/MyD88-dependent signaling activation in CD8^+^ T cells can promote cellular glycolysis and enhance T cell effector functions (142). TLR3 is constitutively expressed on CD8^+^ T effector cells. Furthermore, the TLR3 agonist polyinosinic-polycytidylic acid [Poly (I:C)] increases IFN-γ production in Ag-specific CD8^+^ T cells (143). Poly (I:C) treatment significantly increases the IL-2 and IFN-γ production of chimeric antigen receptor-modified T (CAR T) cells along with improving their lytic action against tumor or cancer cells (144). CAR T cells also show an increased anti-tumor action against refractory or relapsed B cell acute lymphoblastic leukemia upon co-stimulation with TLR2 signaling by introducing the TIR domain of TLR2 into the CAR construct (145). The third-generation anti-CD19 CAR T cells incorporated with the intracellular signaling domains of CD28 and TLR2 are under clinical trial for relapsed or refractory B-cell non-Hodgkin’s lymphoma (146).

## TLR-Mediated Regulation of Regulatory T Cells

Treg cells are critical for maintaining peripheral tolerance, preventing autoimmune diseases, and limiting chronic inflammatory diseases by suppressing host immune responses and inducing self-tolerance (147). CD4^+^ Treg cells are a small subset (5–6%) of the overall CD4^+^ T cell population (121). Foxp3 is a specific marker of CD4^+^ Treg cells in both mice and humans (148–152). In previous studies, the elevated proportion of CD4^+^ CD25^+^ Treg cells in the total CD4^+^ T cell population was observed in several different human cancers, including lung, breast, and ovarian tumors (153–155). We also demonstrated the presence of antigen-specific CD4^+^ Treg cells at tumor sites (152, 156). We showed that Treg cells could suppress the proliferation of naive CD4^+^ T cells and inhibit IL-2 secretion of CD4^+^ effector cells upon activation by tumor-specific antigens (157). In addition, we identified CD8^+^ Treg and γδ-TCR Treg cells in prostate and breast cancer (158, 159). Notably, the CD8^+^ Treg cells expressed Foxp3 molecules, while the γδ-TCR Treg cells did not. Like CD4^+^ Treg cells, both of these CD8^+^ and γδ-TCR Treg cell subtypes have immune suppression ability and inhibit anti-tumor immunity.

To abrogate Treg cell-mediated immune suppression, we sought to identify the TLR ligands that could reverse Treg cell suppressive activity. We found that Poly-G10 oligonucleotides can directly change their suppressive function in the absence of DCs. The TLR8-MyD88 signaling pathway is required to reverse Treg cell function by Poly-G oligonucleotides (158, 160). Moreover, we found that the natural ligands for human TLR8—ssRNA40 and ssRNA33, which are derived from HIV viral sequences (161)—could completely reverse the suppressive function of Treg cells, indicating that activation of the TLR8-dependent signaling pathway is critical for the reversal of Treg-suppressive functions. Besides different subsets of CD4^+^ Treg cells, we found that the CD8^+^ Treg cells and γδ-TCR Treg cells in prostate and breast cancer also express a low level of human TLR8 molecules (158, 159). Interestingly, we demonstrated that Poly-G oligonucleotide treatment could also reverse the suppressive function of CD8^+^ Treg cells and γδ-TCR Treg cells, suggesting that these cells might share the same TLR8/MyD88 signaling pathway-mediated mechanism with previously characterized CD4^+^ Treg cell subsets (158, 159).

Recent studies show that TLR8 stimulation in humans reverses Tregs’ immunosuppressive function and enhances their anti-tumor function by inhibiting glycolysis and glucose uptake (162). CD4^+^ T cells stimulation with TLR8 ligand ssRNA 40 in a co-culture system with ovarian cancer cells (SKOV3) inhibited the glycolysis metabolism and downregulated the percentage of Treg cells (163). Therefore, the TLR8 signaling pathway may regulate Treg by reprogramming the glycolysis metabolism. These findings raise an intriguing possibility that the activation of the TLR8 signaling pathway could block the suppressive function of different subsets of Treg cells to improve the efficacy of cancer immunotherapy.

Since TLR8 is non-functional in mice (164), Poly-G oligonucleotides cannot reverse the suppressive activity of murine Treg cells. However, recent studies showed that other TLR signaling in mice could also mediate the regulation of Treg cells. TLR2-deficient mice showed a reduced number of CD4^+^ CD25^+^ Treg cells (165). Additionally, stimulation of mouse Treg cells with TLR2 ligand Pam3Cys increased its proliferation and temporarily reversed its suppressive function (166, 167). The activation of TLR9 signaling has been reported to inhibit the immunosuppressive function of Treg through direct MyD88-dependent costimulation of effector CD4^+^ T cells (168). However, another study showed that human CD4^+^ CD25^+^ Treg or effector Th1 and Th2 cells did not highly express TLR9 naturally, but 25-dihydroxyvitamin D3 (1α25VitD3)—the active form of Vitamin D—could induce it (169). Stimulation of 1α25VitD3-induced IL-10–secreting Treg with TLR9 agonists showed a decreased IL-10 and IFN-γ production, indicating the reduction of their immunoregulatory function (169). In contrast, stimulation of human Treg cells with the TLR5 ligand flagellin increased rather than reversed their suppressive function (170). The TLR4 ligand LPS was also reported to induce proliferation and enhance the suppressive function of Treg cells (171).

## Regulators in TLR Signaling

Uncontrolled TLR signaling activation can be harmful or even fatal (172). Therefore, the stringent and precise regulation of TLR signaling pathways is essential to maintaining immune balance in the host. In the last few years, many positive and negative regulators have been identified to control TLR-induced NF-κB signaling pathways at multiple levels through different mechanisms (173). These regulators include co-receptors, such as CD14 (174, 175); soluble receptors, such as sTLR (176, 177); transmembrane proteins, such as ST2L (178), SIGIRR (179, 180), and TRAILR (181); and intracellular regulators, such as SOCS-1 (182, 183), MyD88s (184, 185), TOLLIP (186), IRAK-M (187), A20 (188, 189), CYLD (the familial cylindromatosis tumor suppressor gene) (190–194), Nrdp1 (195), regulatory Nod proteins (196–206), TRIAD3A (207), and tripartite motif-containing proteins (TRIMs) (208). These molecules maintain the balance between activation and inhibition of TLR signaling in response to diverse PAMPs (172).

We also participated in the identification of some critical regulators of TLRs and the NF-κB signaling pathway. NLRs were originally believed to function as pathogen sensors and cellular danger signals. However, alongside other groups, we recently found that several NLRs, known as regulatory NLRs, negatively regulate TLR and RIG-I-like receptor signaling. NLR family member X1 (NLRX1) is the first NLR that was identified to negatively modulate RIG-I-mediated antiviral responses by binding to mitochondrial antiviral-signaling protein (MAVS) and disrupting RIG-I-MAVS (209). Then, we found that it could also negatively regulate TLR-induced NF-κB signaling by targeting the TRAF6 and IKKα/β-NEMO complex (199, 201). Besides NLRX1, NLR family CARD domain containing 5 (NLRC5) is another member of the NLR protein family that is recognized as a novel regulator of both adaptive and innate immune responses (210). We identified NLRC5 as a negative regulator of both NF-κB and Type I IFN signaling (196, 200, 202, 203, 206). NLRC5 inhibits IKK phosphorylation and NF-κB signaling by interacting with IKKα/β but not NEMO. NLRC5 inhibits Type I IFN signaling by targeting RIG-I/MDA5 after viral infection and blocking the RIG-I–MAVS interaction. We recently identified NLR family pyrin domain-containing 11 (NLRP11) as a regulatory NLR to attenuate TLR signaling by targeting TRAF6 for degradation *via* the ubiquitin ligase RNF19A (205).

Besides the NLR family, we also discovered some regulators from the LRR-containing (LRRC) family, ubiquitin-specific protease (USP) family, and tripartite motif family (TRIM) family. We found that LRRC25 negatively regulates the TLR-induced NF-κB signaling pathway by promoting p65/RelA for autophagic degradation (211). Interestingly, LRRC25 also inhibits Type I IFN signaling by targeting IFN-stimulated gene 15 (ISG15)-associated RIG-I for autophagic degradation (212). We found that USP38 could also negatively regulate TLR and RIG-I signaling through different mechanisms (213, 214). In contrast, TRIM14 functions as a positive regulator in the noncanonical NF-κB signaling pathway and cGAS- and RIG-I-mediated Type I IFN signaling pathway (215–218).

## TLR-Mediated Immunity in Cancer

Deidier observed that patients infected with syphilis had remission of malignant tumors, revealing the correlation between immune system activation triggered by infection and cancer remission (219). Studies on TLRs involved in cancer have shown that TLR signaling has not only anti-tumor effects but also pro-tumor functions on carcinogenesis, which is dependent on the individual TLR and cancer type (220, 221). TLR stimulation enhances the anti-tumor immune response either through immune cells or directly targeting tumor cells to induce apoptosis. In murine models of hepatocellular carcinoma, TLR2-deficient mice showed a decrease in the expression of IFN-γ, TNF-α, (IL)-1α/β, IL-6, and Cxcl-2, which attenuate p21- and p16/pRb-dependent senescence, leading to the increased proliferation of tumor cells (222). We found that TLR8 ligand treatment suppresses prostate and breast cancer by reversing the function of CD8^+^ Treg cells and γδ-TCR Treg cells (160). Shanshan Qi et al. (223) generated hTLR8 mice by replacing exon 3 of mouse *Tlr8* with human *TLR8* to analyze the role of TLR8 in tumor progression. They found that the MC38 tumor grew slower in hTLR8 mice compared with naïve mice. hTLR8 mice also exhibit increased IFN-γ and TNF-α positive CD4^+^ T cells and effector T cells (223).

In addition, a synthetic bacterial lipoprotein (a TLR1/TLR2 agonist) was reported to reduce the suppressive function of Foxp3^+^ Treg cells and enhance the cytotoxicity of tumor-specific CTL (224). Combination treatment with the TLR1/2 ligand Pam_3_CSK_4_ and anti-CTLA4 mAb improved the anti-tumor immunity compared with anti-CTLA4 mAb alone. This study showed that TLR1/2 increased FcgR IV expression in macrophages, which led to Treg cell depletion and augmentation of T cell/Treg ratios within the tumor (225). Besides their anti-tumor effects, TLRs have also shown pro-tumor functions. Stimulation of TLR4 by LPS promoted immunosuppressive cytokine production, resulting in tumor immune evasion in lung cancer cells (226). In breast cancer, a stimulation expressed-TLR4 tumor with LPS promoted cancer cell proliferation *via* upregulation of IL-8 and IL-6 production (227, 228). Interestingly, TLR6 signaling was recently reported to prevent the inflammation by impacting the composition of microbiota during inflammation-induced colorectal cancer (229). Besides TLRs, MyD88 is also involved in cancer development. MyD88-dependent signaling is reported to control the expression of several key modifier genes of intestinal tumorigenesis and play a crucial role in both spontaneous and carcinogen-induced tumor development (230). Besides, diethylnitrosamine (DEN) administration induced higher serum interleukin-6 (IL-6) production in males than it did in females in DEN-induced hepatocellular carcinoma model. Further study showed that DEN exposure promoted the production of IL-6 in Kupffer cells (KCs) in a MyD88-dependent manner and depletion of MyD88 protected male mice from DEN-induced hepatocarcinogenesis (231). In the activated B-cell-like (ABC) subtype of diffuse large B-cell lymphoma (DLBCL), MyD88 L265P is reported to contribute to the constitutive NF-κB and JAK kinase signaling, which promotes malignant cell survival in these lymphomas (232). MyD88 L265P somatic mutation is identified as a commonly recurring mutation in patients with Waldenström’s macroglobulinemia (233). 69% of patients with cutaneous diffuse large B cell lymphoma (CBCL) carry MyD88 L265P mutation, which is significantly associated with shorter disease-specific survival (234). In addition, the MyD88/IL1 receptor (IL1R) axis upregulates programmed cell death (PD)-1 expression on tumor-associated macrophages (TAMs) *via* promoting recruitment of NF-κB to the Pdcd1 promoter, which sustains their immunosuppressive function in melanoma (235). Based on the critical role of TLRs and TLRs-mediated signaling pathways in cancer development, researchers have taken advantage of agonists and antagonists of TLRs to treat some types of cancer **(** **Table 1** **)**. Various agonists of TLRs are currently under investigation in clinical trials for cancer treatments **(** **Table 2** **)**. Due to the double-edged role of TLRs in tumor biology, it is essential to understand how TLRs manipulate the immune system and tumor cell characteristics, which may provide us with new therapeutic strategies against cancer.

**Table 1:** TLR agonists, antagonists and cancer.

TLR	Agonist/Antagonist	Cancer and Model	Observation	Reference
**TLR1/2**	Bacterial lipoprotein, Pam3CSK4	Lung carcinoma, leukemia, and melanoma	Inhibits the suppressive function of Foxp3+ Tregs and enhance the cytotoxicity of tumor-specific CTL; depletion of tumor-infiltrating Treg cells	(224, 225)
**TLR2/TLR4**	OM-174 (synthetic derivative of lipid A), bacille Calmette-Guérin (BCG)	Melanoma, bladder cancer	Increases natural killer cell and CTL activity; prolongs survival of bladder cancer patients	(236, 237)
**TLR3**	Poly I:C, poly-ICLC(Hiltonol)	B16 melanoma cells, facial embryonal rhabdomyosarcoma	IFN-γ plus poly I:C reduces the expression of PD-L1; shows tumor regression and prolonged survival	(238, 239)
**TLR4**	MPLA	Breast and ovarian cancer models	MPLA + IFNγ repolarizes TAMs to tumoricidal macrophages and activates cytotoxic T cells	(240)
**TLR4**	TAK-242 (resatorvid), Eritoran	Breast cancer, colorectal cancer	Inhibits breast cancer cell viability, inhibits the proliferation of breast cancer cells, induces G2/M cell cycle arrest in breast cancer cells and induces apoptosis of breast cancer cells; blocks LPS-enhanced-AKT phosphorylation in colorectal cancer cells	(241, 242)
**TLR5**	Entolimod	Murine colon and mammary metastatic cancer models	Restrains liver metastases and facilitates the formation of CD8^+^ T cell memory	(243)
**TLR7**	Imiquimod	Various cutaneous malignancies	Induces apoptosis, induces production of various cytokines, and stimulates cell-mediated immune response	(244)
**TLR7/8**	MEDI9197	B16-OVA melanoma tumor model	Localized administration of TLR7/8 agonism polarizes anti-tumor immunity towards a Th1 response and activates natural killer cells and CD8^+^ T cells	(245)
**TLR7/TLR9**	Chloroquine	Hepatocellular carcinoma	Downregulate the level of phosphoralated-AKT and inhibit HuH7 cell proliferation;	(246)
**TLR9**	CpG	Colon cancer animal model, head and neck cancer animal model, melanoma	Reverses resistance to PD-1 blockade therapy by expending CD8^+^ T cells; enhances the efficacy of anti-PD-1 therapy; expands tumor antigen-specific CD8^+^ T cells	(247–249)

**Table 2:** Clinical trials of TLR agonists TLR in cancer.

TLR	TLR agonist	Cancer type	Status	Reference
**TLR3**	poly-IC12U (Ampligen)	Colorectal cancer	Phase II	NCT04119830 NCT03403634
		Melanoma	Phase II	NCT04093323
		Prostate cancer	Phase II	NCT03899987
	poly-ICLC (Hiltonol)	Non-Hodgkin’s Lymphoma, breast cancer, head and neck squamous cell carcinoma	Phase I/II	NCT03789097
		Melanoma	Phase I/II	NCT03617328
		Mesothelioma	Phase I	NCT04525859
		Prostate cancer	Phase I	NCT03835533
**TLR4**	MPLA	Melanoma, ovarian cancer, lung cancer	Phase I/II	NCT01584115
	GLA-SE	Stage III adult soft tissue sarcoma, stage IV adult soft tissue sarcoma	Phase I	NCT02180698
		Follicular low grade non-Hodgkin’s lymphoma	Phase I/II	NCT02501473
**TLR5**	Mobilan	Prostate cancer	Phase I	NCT02844699
	Entolimod	Advanced or metastatic solid tumors cancers	Phase I	NCT01527136
**TLR7**	Imiquimod	Superficial basal cell carcinoma	Phase III	NCT00189306
		Malignant melanoma	Phase I	NCT00142454
		High-risk melanoma	Phase II	NCT00273910
**TLR7/8**	Resiquimod	Stage II, Stage III, or Stage IV Melanoma	Phase I	NCT00470379
**TLR9**	MGN1703	Metastatic colorectal carcinoma	Phase III	NCT02077868
	SD-101	Non-Hodgkin lymphoma	Phase I	NCT03410901

## TLR-Mediated Immunity in Autoimmune Disease

TLRs are supposed to sense pathogenic components and initiate the immune response that contributes to host homeostasis. However, in the specific scenario, TLRs are improperly activated by self-antigens, leading to chronic systemic inflammatory disorders and the occurrence of autoimmunity. Numerous studies have demonstrated that TLRs are involved in the pathogenesis of various autoimmune diseases such as rheumatoid arthritis (RA), systemic lupus erythematosus (SLE), multiple sclerosis, and Crohn’s disease (250) **(** **Table 3** **)**. RA is an autoimmune disorder that affects the synovial joints, causing chronic and persistent inflammation and the destruction of articular tissues. MyD88 also has been demonstrated to be crucial for the production of MMPs (the major enzymes involved in joint tissue destruction) in RA synovial membrane cultures (275). TLR2 and TLR4 expression were reported to be associated with the levels of IL-12 and IL-8 in the synovial tissue of RA patients (251). The surface expression of TLR4 on CD8^+^ T cells directly correlates with the disease severity of RA. And the TLR4-expressing CD8^+^ T cells can respond to LPS and express robust amounts of cytolytic and inflammatory molecules including TNFα and IFNγ (256). Besides RA, TLR2 and TLR4 are also involved in heat shock proteins-associated atherosclerosis (257, 258). Moreover, emerging evidence indicates that TLR2 is strongly associated with diabetes (259–261).

**Table 3:** TLRs implicated in autoimmune diseases.

Autoimmune diseases	TLR	Reference
**Rheumatoid arthritis**	TLR2, TLR4, TLR3/7, TLR9	(251–256)
**Atherosclerosis**	TLR2, TLR4	(257, 258)
**Diabetes**	TLR2	(259–261)
**Systemic lupus erythematosus**	TLR7, TLR8, TLR9	(262–268)
**Systemic sclerosis**	TLR2, TLR8	(269–271)
**Myositis**	TLR3, TLR4, TLR7, TLR9	(272–274)

SLE is characterized by the presence of autoantibodies triggered by CpG DNA and ssRNA-associated self-antigens. In endosomes, the self-antigens are sensed by TLR7 and TLR9. TLR7 is essential for generating the germinal center and drives the extrafollicular pathway, which is associated with pathogenic antibody secretion. Notably, TLR9 has been demonstrated to have a protective function in SLE by limiting the stimulatory activity of TLR7 (262). Genetic studies have shown that copy number variation of TLR7 is associated with SLE development (263, 264). Additionally, TLR7 localizes on the X chromosome escapes X inactivation in B cells and myeloid cells in females, resulting in the gender difference in TLR7 expression (265), leading to a higher incidence in women than men. Moreover, SLE patients with increased expression of TLR7 showed significant expansion of CD19^+^ IgD^+^CD38^++^ transitional B cells and increased IgG auto-Ab production (266). Recent data show that the expression of *TLR7* in mild and severe lupus-prone models is dependent on the activity of IRAK4 (the TLR7-downstream signaling molecule) and the pathogenic environment. Impairments of IRAK4 signaling refrain from all pathological characteristics associated with murine lupus. These data suggest a feedback loop of TLR7 expression and pathological changes in SLE patients (267).

A study in experimental autoimmune encephalomyelitis (EAE) mice models showed that deficiency of MyD88 conferred complete resistance to EAE in mice, indicating that a TLR-mediated immune response is required to induce EAE (276). Consistently, depletion of TLR4 solely in CD4^+^ T cells impairs Th17 and markedly abolishes the disease symptoms (135). In addition, accumulating evidence suggests that TLR8 contributes to autoimmune diseases as well. It is reported that human monocytes that lack CD14 (CD14^dim^) and express CD16 do not produce cytokines in response to bacterial cues that are sensed by cell-surface TLRs. Instead, they trigger the production of TNF-α, IL-1β, and C-C motif chemokine ligand 3 in response to viruses through the TLR7-TLR8-MyD88-MEK pathway. Further study showed that these CD14^dim^ monocytes recognize self-nucleic acids and drive the production of inflammatory cytokines in patients with lupus (268).

Cristiana et al. (277) used human *TLR8*-transgenic mice to show that high copy number chimeras developed the multiorgan inflammatory syndrome through DC-intrinsic huTLR8 activation and subsequent T cell activation. The severity of the inflammation was associated with the expression level of huTLR8. They observed spontaneous arthritis in high-expressing human *TLR8* mice. Furthermore, they demonstrated that *TLR8* mRNA expression was much higher in blood from both SoJIA and Still’s disease donors than healthy donors. Finally, the mRNA level of *TLR8* was associated with the transcription level of inflammatory cytokines in these patients.

In addition, the ectopic expression of TLR8 on pDCs in systemic sclerosis patients induces the production of CXCL4, which in turn enhances TLR8- and TLR9-induced IFN production by pDCs. Both CXCL4 and IFNs are the featured cytokines in systemic sclerosis (269). These data suggest that TLR8 is the key RNA-sensing TLR in the pathogenesis of autoimmune disease, demonstrating the potential of TLR8 for clinical development. Emerging evidence suggests numerous autoimmune diseases are triggered by the dysregulation of TLR. Some TLR antagonists have already been applied to autoimmune disease treatment in mice models. Due to redundancy between different TLRs in different disease-affected tissues, it is crucial to dissect the detailed molecular mechanism and cell-mediated immune regulation in the specific disease context to facilitate drug development for clinic treatments.

## TLR-Mediated Immunity in Infectious Disease

TLRs play an essential role in host immune responses to various invading pathogens, including bacteria, fungi, viruses, and parasites **(** **Table 4** **)**. TLR1 is crucial for the induction of mucosal Th17 immunity and IgA responses during *Yersinia enterocolitica* infection (316, 317). The I602S mutant of TLR1 results in the deficiency of TLR1 trafficking from the cytosol to the cell surface, potentially impairing blood monocytes’ immune functions against pathogenic *Mycobacterium tuberculosis* (318). Recently, it was reported that mice deficient in both TLR2 and TLR4 were highly susceptible to intracellular *Salmonella typhimurium* infection (319). However, *Tlr2/4*-deficient mice lacking additional TLR9 involved in *S. typhimurium* recognition were less susceptible to infection (319). Notably, TLR2 was also reported to recognize the envelope (E) protein of SARS-CoV-2 to induce a hyperinflammatory response in mice bone-marrow-derived macrophages (283). Besides TLR2, TLR4 was also reported to recognize the spike (S) protein of SARS-CoV-2 and activate the NF-κB signaling to produce IL-1β (320).

**Table 4:** TLRs and infectious diseases.

TLR	Class of Pathogen Recognized	Infectious Agent	Reference
**TLR1/2**	Bacteria	Mycobacteria	(278–280)
**TLR2**	Bacteria	*Staphylococcus aureus* *Listeria monocytogenes*	(281, 282)
	ssRNA viruses	SARS-CoV-2	(283)
**TLR2/3**	Protozoa	*Neospora caninum*	(284)
**TLR3**	DNA viruses	HSV	(285)
	Retroviruses	HIV	(286–290)
	ssRNA viruses	Respiratory syncytial virus	(291–294)
	Protozoa	*Neospora caninum*	(295)
**TLR4**	Bacteria	*Staphylococcus aureus*	(281, 296)
	ssRNA viruses	Syncytial virus	(297)
		Rabies virus	(298, 299)
	Bacteria	Mycobacteria	(300)
**TLR5**	Bacteria	*Burkholderia pseudomallei*	(301)
**TLR2/6**	ssRNA viruses	Dengue virus	(302)
**TLR6**	Bacteria	*Legionella pneumophila*	(303)
**TLR7**	Protozoa	*Leishmania*	(304)
**TLR7/8**	ssRNA viruses	Influenza A	(305)
	Retroviruses	HIV-1	(306–308)
**TLR8**	Retroviruses	HIV-1	(309)
	Bacteria	*Staphylococcus aureus*	(310)
**TLR9**	DNA viruses	HSV-1, HSV-2	(311)
		HPV	(312)
		Adenovirus	(313–315)

Numerous studies have shown that TLR4 is also involved in various infectious diseases. Infants carrying D299G and T399I polymorphisms are more vulnerable to respiratory syncytial virus infection (321). The single nucleotide polymorphism rs11536889 of TLR4 is involved in organ failure in sepsis patients (322). However, the effects of TLR4 remain controversial during *M. tuberculosis* infection. It was reported that *Tlr4*-deficient mice exhibited the same sensitivity compared to congenic control mice (323). By contrast, another study found that TLR4 mutant mice showed reduced macrophage recruitment and failure to develop a protective immune response against chronic *M. tuberculosis* infection (324). Melioidosis is a high-mortality infectious disease caused by *Burkholderia pseudomallei*, a flagellated, Gram-negative bacterium. TLR5 c.1174C>T (a TLR5 variant carrying a nonsense mutation) is associated with lower IL-10 and TNF-α production and prolonged survival in human melioidosis.

Influenza A virus is a contagious agent that causes respiratory disease. TLR7 is responsible for influenza A virus sensing in the endosome, while RIG-I senses influenza A virus in the cytosol (161, 325–327). The sensing mechanisms for TLR7 and RIG-I are different. TLR7 directly recognizes virus ssRNA in a virus-replication-independent manner. By contrast, RIG-I recognizes the viral replication intermediates in certain cell types (328). Both signaling pathways move toward the activation of IRF3/7 and NF-κB to trigger the production of Type I IFN and proinflammatory cytokines and downstream IFN-stimulated genes (1). Furthermore, it has been shown that intranasal administration of the TLR7 agonist (imiquimod) can significantly reduce airway and pulmonary inflammation in mice during influenza A virus infection (329).

Different single nucleotide polymorphisms of TLR8 and TLR9 confer varying degrees of risk in the development of tuberculosis, suggesting that TLR8 and TLR9 are involved in tuberculosis (330). The most characteristic role of TLRs is sensing the PAMPs from pathogens and initiating immune responses against infectious agents. Notably, in certain scenarios, TLRs may be subverted by the pathogens to alter the host cytokine pattern for their own benefit (319, 331). Thus, further studies on the interplay between pathogen evasion and TLR subversion will have implications for human health.

## Conclusion and Perspectives

**Figure 3:**
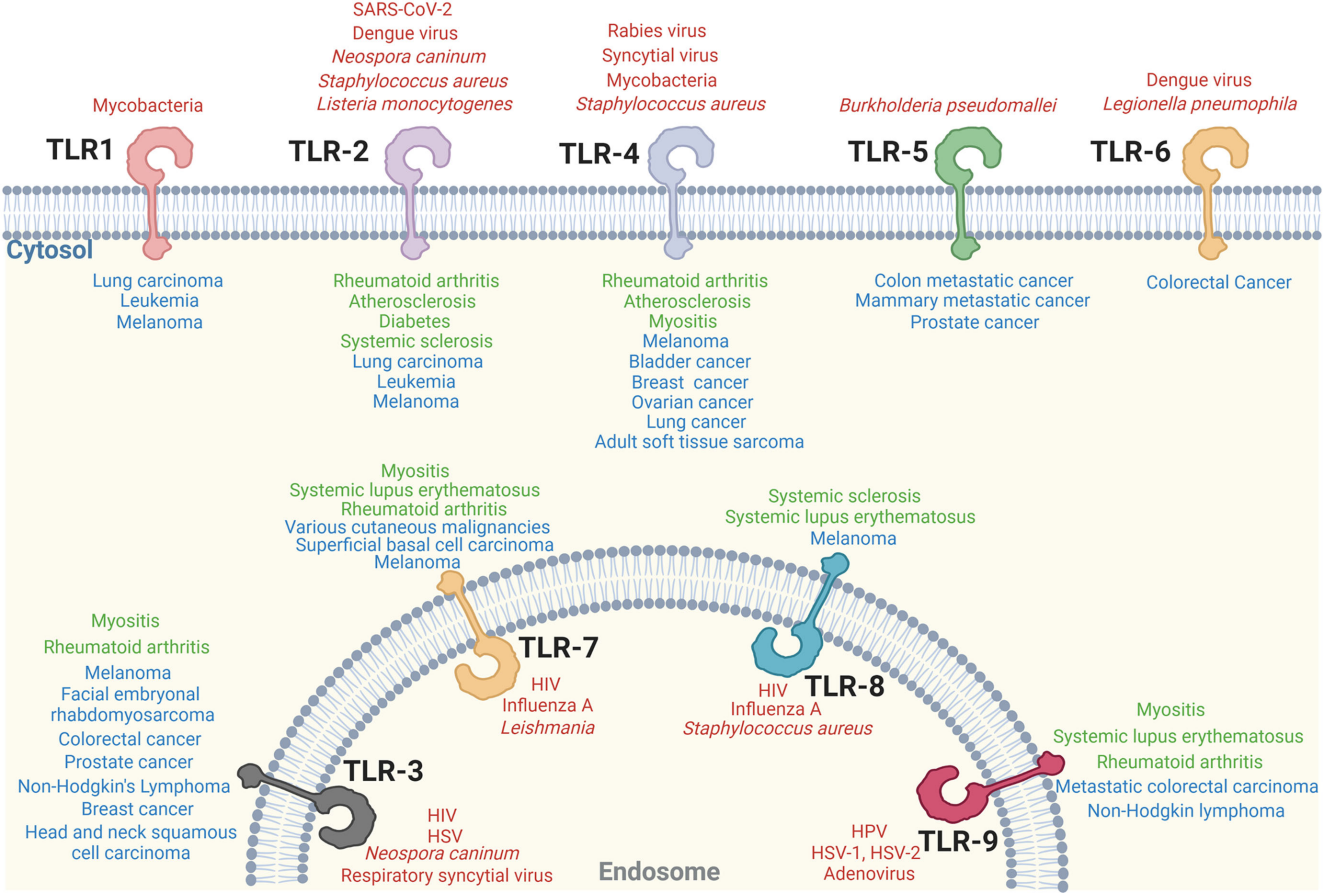
Individual TLR singaling involves in various diseases. Individual TLR-associated infectious diseases (red), autoimmune diseases (green) and cancer (blue) are shown.

This review provides an updated overview of TLR signaling and its critical role in cell-mediated immunity. The fundamental mechanisms of TLR signaling transduction have been identified by cell biological and biochemical approaches, as well as loss-of-function genetic analysis. Significant progress has also been made in the structural elucidation of TLRs and their downstream signaling supramolecular complex (332, 333). The essential role of TLR signaling in activating innate immune cells to initiate adaptive immunity was illustrated. Importantly, the direct regulatory roles of TLR signaling in effector T cells and Treg cells have been identified. In addition, the individual TLRs signaling involved in infectious disease, autoimmune disease and cancer have been extensively studied (**Figure 3**).

Despite the rapid advancement of our knowledge, there are still large gaps in our understanding of TLR signaling. For example, although free unanchored K63-Ub chains have been demonstrated as a kind of indispensable “second messenger” to activate downstream protein kinases during TLR-induced NF-κB signaling activation by biochemical experiments, how and whether these free unanchored K63-Ub chains activate downstream protein kinases in cells is still unknown. Especially, how to control the activation specificity if these chains are just free in the cytoplasm still remains elusive. Therefore, the detailed mechanisms of how these polyubiquitin chains activate the kinase complex during TLR signaling pathway activation warrant further investigation.

Due to the vital role of TLR signaling in T cell activation, growth, differentiation, and function, it would be necessary to dissect the T cell-specific TLR signaling pathway. In the past few years, T cell-based cancer immunotherapy has made significant progress (334). The Food Drug Administration (FDA) has approved four CD19-CAR-engineered T cell products for blood cancer. Cancer vaccines along with TLR signaling activation could become a more effective therapeutic approach to inhibiting or even eliminating cancer cells. Cancer vaccines along with TLR signaling activation could become a more effective therapeutic approach to inhibiting or even eliminating cancer cells (335).

Besides cancer immunity, TLRs are also involved in many infectious diseases by recognizing the PAMPs of pathogens, initiating inflammatory responses, and eliminating invasive microorganisms at the early stage. However, prolonged or excessive inflammatory responses are harmful or even fatal for the host at the late stage. In the current COVID-19 pandemic, fatal hyperinflammation, but not SARS-CoV-2 directly, is the primary cause of mortality in severe COVID-19 patients. Therefore, drugs targeting viral replication might be ineffective for severe COVID-19 patients since most hospitalized patients are at the late stage of disease. In this case, drugs targeting TLR-dependent inflammatory signaling pathways might be more effective in reducing the mortality of severe COVID-19 patients.

As the field has developed, multidisciplinary approaches have been used in the study of TLR signaling pathways. Integrated methods, combined with transcriptomics, genetic/chemical perturbations, and phosphoproteomics, have been used to systematically discover TLR signaling regulatory components. The m6A RNA sequence technique led to the discovery that mRNA stability is an essential mechanism for regulating TLR-dependent innate immune responses. The single-cell sequencing (scRNA-seq) approach is used to dissect the characteristics of TLR-mediated immune responses at the single-cell level. The development of super-resolution single-molecule localization microscopy empowers the ability to directly observe the supramolecular signaling complex during TLR signaling activation at the single-molecule level. Finally, the advances in cryo-electron microscopy have facilitated our understanding how TLRs recognize their ligands and initiate immune signaling at the atom level. These recent advancements markedly increase our ability to understand TLR signaling pathways and develop new therapeutic strategies against various infectious, autoimmune diseases, and cancers.

## Author Contributions

R-FW, TD, and YD designed and wrote the manuscript. TD, YD, CX, HW, and R-FW discussed and revised the manuscript. All authors contributed to the article and approved the submitted version.

## Funding

This work was in part supported by grants from the NCI, NIH (R01CA101795, R01CA246547 and U54CA210181), Department of Defense (DoD) CDMRP BCRP (BC151081) and LCRP (LC200368) to R-FW.

## Conflict of Interest

The authors declare that the research was conducted in the absence of any commercial or financial relationships that could be construed as a potential conflict of interest.

## Publisher’s Note

All claims expressed in this article are solely those of the authors and do not necessarily represent those of their affiliated organizations, or those of the publisher, the editors and the reviewers. Any product that may be evaluated in this article, or claim that may be made by its manufacturer, is not guaranteed or endorsed by the publisher.
